# Improving ALS Molecular Diagnosis Through Functional Assays: Reassessment of a *SOD1* Variant of Uncertain Significance

**DOI:** 10.3390/ijms26157414

**Published:** 2025-08-01

**Authors:** Léa Bedja-Iacona, Arthur Forget, Chloé Boisseau, Sylviane Marouillat, Aleksandra Chudinova, Charlotte Veyrat-Durebex, Claire Guissart, Serge Lumbroso, Cédric Raoul, Christian R. Andres, Hélène Blasco, Philippe Couratier, Philippe Corcia, Annie Verschueren, Kevin Mouzat, Patrick Vourc’h

**Affiliations:** 1 Université de Tours, INSERM, Imaging Brain & Neuropsychiatry iBraiN U1253, 37032 Tours, France; 2 INM, Univ Montpellier, INSERM, 34000 Montpellier, France; 3 CHU de Tours, Service de Biochimie et Biologie Moléculaire, Laboratoire de Biologie Médicale de Référence (LBMR) sur la SLA, 37044 Tours, France; 4 Laboratory of Biochemistry and Molecular Biology, Nimes University Hospital, 34295 Montpellier, France; 5 CHU de Limoges, Centre de Référence sur la SLA de Limoges, 87100 Limoges, France; 6 CHU de Tours, Centre de Référence sur la SLA de Tours, 37044 Tours, France; 7 CHU de Marseille, Centre de Référence pour les Maladies Neuromusculaires et la SLA, 13005 Marseille, France

**Keywords:** ALS, models, neurogenetics, variants, SOD1

## Abstract

Genetic testing in amyotrophic lateral sclerosis (ALS) often reveals variants of uncertain significance (VUS), which are frequently omitted from diagnostic reports or reported with limited clinical interpretation. To address this gap, we developed a rapid functional assessment pipeline in collaboration with FILSLAN, the French ALS care network, combining in vitro and in vivo neurogenetic assays. We illustrate this approach through the reclassification of the *SOD1* p.Val120Leu variant, identified in an ALS patient, as pathogenic. Functional studies demonstrated that this variant leads to cytoplasmic aggregation, reduced neurite outgrowth, and abnormal motor behavior in zebrafish. These results support the systematic use of functional assays to clarify the pathogenicity of uncertain variants, thereby improving diagnostic accuracy, preventing misdiagnosis, and enabling timely therapeutic interventions in ALS.

## 1. Introduction

Amyotrophic lateral sclerosis (ALS) appears in 15% of cases as a monogenic, mainly autosomal dominant disease. To date, thirty genes responsible for ALS have been identified [1,2]. The identification of a pathogenic variant in a patient provides crucial information, both medically and scientifically. It helps to confirm the clinical diagnosis or to exclude other diseases with similar symptoms, to offer patients more personalized follow-up and therapeutic management, and to provide genetic counseling for family members. The NGS technology used for molecular diagnosis in ALS reveals an important number of rare genetic variants in the coding regions of genes studied by targeted analysis, or by exome or genome studies. The classification of these genetic variants by most geneticists is based on the ACMG guidelines [3], which define the following five categories: pathogenic, likely pathogenic, benign, likely benign, and variants of uncertain significance (VUS). Molecular diagnostic laboratories frequently encounter difficulties in interpreting the degree of pathogenicity of genetic variants identified in ALS patients. In such cases, either the variant is reported to the clinician as likely pathogenic, leaving pathogenicity in doubt or it is not reported, being classified as VUS. Many bioinformatic tools exist to predict whether genetic variants are pathogenic, but their performance is still imperfect [4]. Experimental approaches remain essential to evaluate the impact of a variant on gene expression or function. They are also important for studying their consequences at the cellular level (integrity and metabolism, for example), and even at the organismal level. The contribution of in vitro and in vivo models providing functional data on these variants is therefore crucial for characterizing and interpreting their pathogenicity [5].

Here, using the example of a variant identified in a French ALS patient and classified with conflicting pathogenicity interpretations in existing databases, we describe the interpretative workflow we developed in collaboration with the FILSLAN network (French Rare Disease Health Network for ALS and other motor neuron diseases). This approach aims to support the interpretation of variants in an ALS gene, such as *SOD1*, one of the major genes associated with ALS, which currently has more than 130 missense variants. According to the Genebe database, the number of pathogenic and probably pathogenic variants in the *SOD1* gene are 37 and 61, respectively. This gene also contains 56 VUS variants, which currently could not be interpreted and therefore were not returned to patients if pathogenic, resulting in a loss of information for the family and possibly a loss of access to therapeutics.

## 2. Case Presentation

### 2.1. Identification of a Genetic Variant in an ALS Patient

We report a missense heterozygous variant in *SOD1* gene (c.G358C) in a patient with ALS, leading to a valine-to-leucine transition at position 120 of the superoxide dismutase 1 (p.Val120Leu). No other variants were identified in *C9orf72* or in other ALS genes (panel of 30 genes [6]). The patient presented at the age of 40 with isolated cramps and fasciculations, with no family history. At the age of 48, he developed weakness of the lower limbs with progressive atrophy of the thighs, later affecting the distal upper limbs. Electromyography showed chronic diffuse denervation with numerous fasciculations in the cervical and lumbosacral regions. Cerebrospinal fluid and blood tests were normal. Genetic tests were conduct in a clinical care context, in accordance with French regulations governing the examination of genetic characteristics for medical purposes (French Public Health code, art.L1131-1). They were performed after obtaining informed consent, in a laboratory accredited by the French Agence de Biomedecine.

Genetic tests for *SMN1* and Kennedy’s disease were negative. His condition slowly worsened. After 23 years, he had proximal and distal amyotrophy of all limbs, pathological hyperreflexia of the upper limbs and patellar reflex, but no ankle reflex. Bulbar and respiratory functions remained normal, with no cognitive impairment.

### 2.2. Functional Analysis of the Genetic Variant

**Figure 1:**
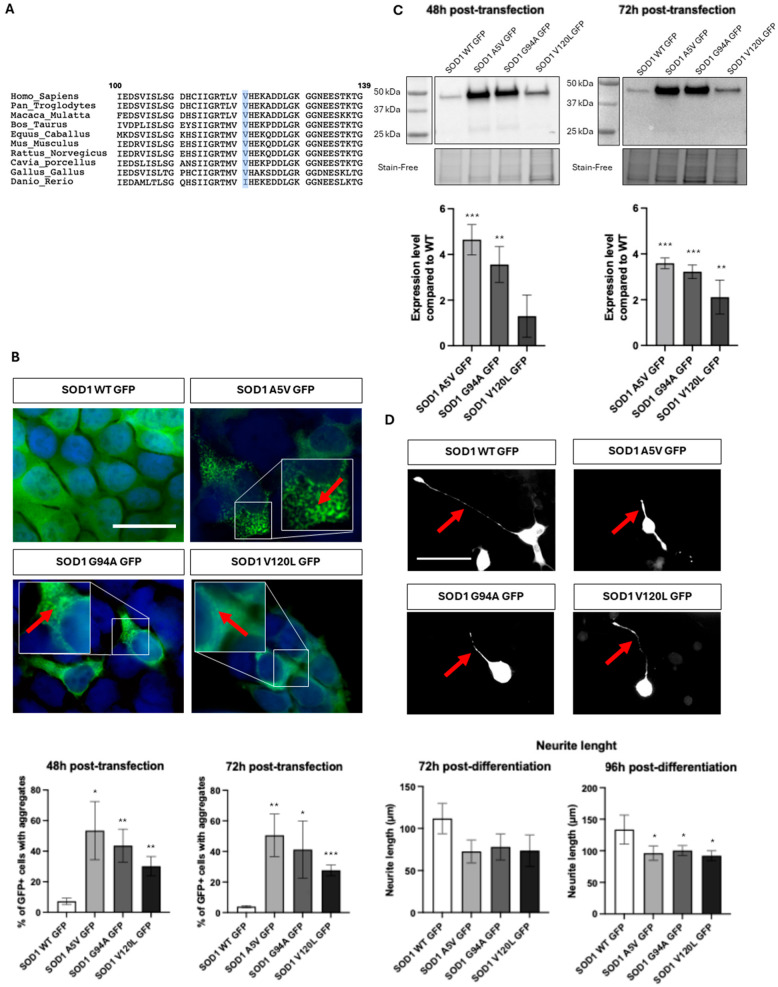
(**A**) Conservation of the Val120 position in SOD1 among animal species using SeaView. (**B**) Immunostaining of HEK293T cells using the GFP antibody (#632592, Takara, Otsu, Japan) 72 h after transfection with various plasmids (Lipofectamine 2000, Thermo Fisher Scientific, Waltham, MA, USA), and percentage of cells showing aggregates 48 or 72 h post-transfection. Plasmids expressed GFP-linked wild-type SOD1 protein (SOD1 WT GFP), the positive controls SOD1 p.Ala5Val (SOD1 A5V GFP), or SOD1 p.Gly94Ala (SOD1 G94V GFP) often used in ALS studies, or the variant of interest SOD1 p.Val120Leu (SOD1 V120L GFP). Red arrows in the enlargements indicate the presence of GFP-positive aggregates. (**C**) Western blot analysis of the insoluble protein fraction in HEK293T cells at 48 and 72 h post-transfection using anti-GFP antibody (log2 fold changes; upper part) (stain-free gel for normalization; bottom part). (**D**) Quantification of neurite length on NSC34 cells 72 h (red arrows on photographs) and 96 h post-differentiation using ImageJ software v2.14.0. *t*-test and Kruskal–Wallis test (* *p* < 0.05; ** *p* < 0.01; *** *p* < 0.001) (scale bars: 75 μm).

Following the ACMG recommendations, we have set up a process to study genetic variants in ALS genes. Interpretation of the p.Val120Leu variant of SOD1 indicates the following. Concerning population analysis, the variant shows a rare allele frequency in control population (1.10-6 in gnomAD). It has been reported in three other ALS cases to date [7,8]. Concerning computational and predictive data, another missense variant at this same position, p.Val120Phe was identified in an ALS patient [9]. Valine in position 120 is conserved in many animal species, but not in the zebrafish *Danio rerio* (Ile120) (Figure 1A). Pathogenicity prediction tools Mutation Taster and Sift predict a damaging character for the variant, but Polyphen does not. Concerning functional data, we performed in vitro and in vivo studies. Expression of SOD1 p.Val120Leu fused to the green fluorescent protein (GFP) in HEK293T cells was associated with an increased number of cells with protein aggregates at 48 and 96 h (*p* < 0.01) (Figure 1B) and higher accumulation of SOD1 protein in the insoluble fraction at 72 h (*p* < 0.01) (Figure 1C). Expression of SOD1 p.Val120Leu in NSC34 motor neurons showed a significant reduction in neurite length at 96 h post-differentiation (*p* < 0.05) (Figure 1D).

**Figure 2:**
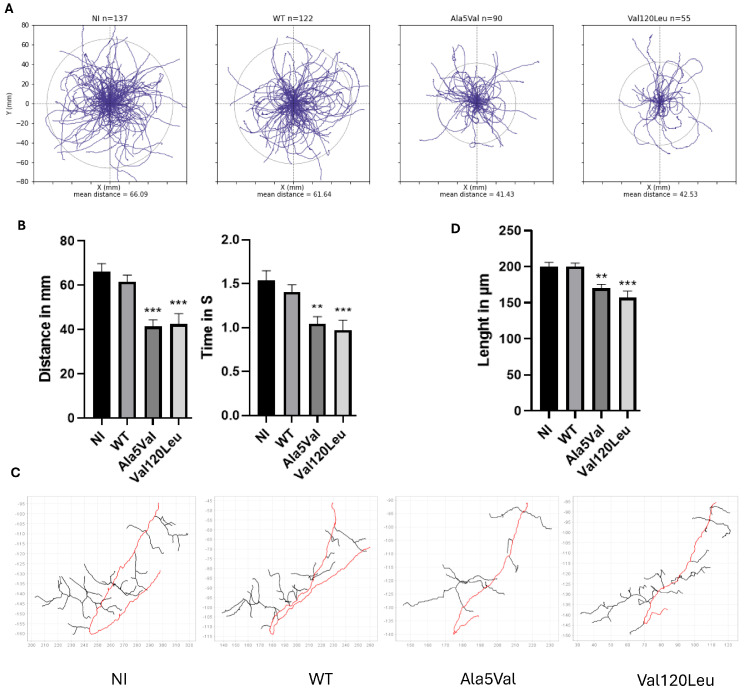
(**A**) Swimming trajectories of zebrafish populations. The different populations studied (n represent the numbers of zebrafish shown on graphs) as follows: negative controls non-injected (NI) or expressing wild-type SOD1 protein (WT), positive controls expressing mutant SOD1 p. Aal5Val, zebrafish of interest expressing SOD1 p.Val120Leu variant. Circles represent the average distance of each population. (**B**) Distance traveled and swimming time for each zebrafish (** *p* < 0.01; *** *p* < 0.001). (**C**) Schematic representation of zebrafish CAP motoneurons from confocal images. Motor neurons were labeled with anti SV2 primary antibody and axonal projections (red) were reconstructed based on Z-stack images. NI (Non-Injected) and WT (Wild-type) were used as negative controls and p.Ala5Val injected embryos were used as positive control. (**D**) Average axonal length of motoneurons for each group (mean ± standard error means). N = 10 larvae for each group (** *p* < 0.05; *** *p* < 0.01).

We next used the zebrafish model, an in vivo model often used in ALS studies [10]. Zebrafishes expressing the SOD1 p.Val120Leu variant showed behavioral swimming deficits (reduced swimming distance and time) and decreased axonal length similar to zebrafish expressing the pathogenic mutant SOD1 p.Ala5Val (*p* < 0.01), a well-characterized positive control in ALS (Figure 2A–C).

## 3. Discussion

The clinical diagnosis of ALS is now routinely accompanied by molecular genetic testing, most commonly via NGS panels targeting genes known to be implicated in the disease. While this approach has significantly improved our ability to detect potentially causative variants, it also generates a high number of variants of uncertain significance (VUS), or variants classified as likely pathogenic according to ACMG guidelines, which require further evaluation before clinical interpretation [11]. In some cases, VUS are not reported to clinicians at all, limiting the potential for timely and informed medical decisions, especially in familial forms of ALS or in cases where treatments may be considered. To address this challenge, we have developed, in collaboration with the French FILSLAN ALS care network, a rapid and integrative pipeline for the evaluation of these ambiguous variants. This pipeline leverages standard tools available in most diagnostic laboratories, including segregation analysis, population frequency data, and in silico pathogenicity prediction algorithms. However, we have complemented this pipeline with additional in vitro and in vivo functional assays, which remain underutilized in routine diagnostic workflows. Functional assays generate empirical data on the molecular and cellular consequences of a variant, providing critical information that enhances and validates in silico or statistical interpretations.

Our study demonstrates the feasibility and clinical utility of incorporating functional data into variant interpretation. The reclassification of the *SOD1* p.Val120Leu variant, initially considered of uncertain significance, as pathogenic based on consistent functional evidence (including protein aggregation, altered neuronal morphology, and abnormal behavior in zebrafish), demonstrating the added diagnostic value of functional approaches. These findings support the broader integration of functional genetics into diagnostic pipelines, especially in neurodegenerative diseases like ALS, where genetic heterogeneity and phenotypic variability complicate interpretation [12,13]. Given that co-segregation analysis is not possible in sporadic forms of ALS, it is particularly important to perform functional studies on the identified variants. Although limitations exist, such as protein overexpression in cellular models or the phylogenetic distance between zebrafish and humans, these models have shown strong predictive value when tested on well-characterized pathogenic variants, including *SOD1* p.Gly94Ala and p.Ala5Val [14,15]. This supports their relevance and robustness as tools for variant interpretation.

## 4. Conclusions

The systematic use of functional validation may not only improve diagnostic accuracy but also reduce diagnostic delays and avoid misclassification, which can have direct implications for patient management and genetic counseling. Future efforts should focus on standardizing functional testing protocols and incorporating them into ACMG-based classification systems to support their broader adoption in a clinical genomics. Given the growing number of variants in ALS-associated genes that remain classified as VUS or likely pathogenic under ACMG guidelines, there is a pressing need for diagnostic laboratories to incorporate simple, rapid, and reliable functional neurogenetic assays into their workflows. These tools can provide actionable data in clinically relevant periods, which is increasingly important in an era of emerging therapeutic options in ALS.

## Data Availability

The raw data supporting the conclusions of this article will be made available by the authors on request.

## References

[1] Pottinger T.D., Motelow J.E., Povysil G., Moreno C.A.M., Ren Z., Phatnani H., Aitman T.J., Santoyo-Lopez J., New York Genome Center ALS Sequencing Consortium, Scottish Genomes Partnership (2023). Rare Variant Analyses Validate Known ALS Genes in a Multi-Ethnic Population and Identifies ANTXR2 as a Candidate in PLS. MedRxiv.

[2] Yuan L., Yang Y., Guo Y., Deng H. (2025). Genetic architecture of amyotrophic lateral sclerosis: A comprehensive review. J. Genet. Genom..

[3] Richards S., Aziz N., Bale S., Bick D., Das S., Gastier-Foster J., Grody W.W., Hegde M., Lyon E., Spector E. (2015). Standards and Guidelines for the Interpretation of Sequence Variants: A Joint Consensus Recommendation of the American College of Medical Genetics and Genomics and the Association for Molecular Pathology. Genet. Med..

[4] Ghosh R., Oak N., Plon S.E. (2017). Evaluation of in Silico Algorithms for Use with ACMG/AMP Clinical Variant Interpretation Guidelines. Genome Biol..

[5] Rodenburg R.J. (2018). The functional genomics laboratory: Functional validation of genetic variants. J. Inherit. Metab. Dis..

[6] Corcia P., Camu W., Brulard C., Marouillat S., Couratier P., Camdessanché J.-P., Cintas P., Verschueren A., Soriani M.-H., Desnuelle C. (2021). Effect of Familial Clustering in the Genetic Screening of 235 French ALS Families. J. Neurol. Neurosurg. Psychiatry.

[7] de Fuenmayor-Fernández de la Hoz C.P., Hernández-Laín A., Olivé M., Arteche López A., Esteban J., Domínguez-González C. (2020). SOD1 Mutations in Adult-Onset Distal Spinal Muscular Atrophy. Eur. J. Neurol..

[8] Nunes Gonçalves J.P., Leoni T.B., Martins M.P., Peluzzo T.M., Dourado M.E.T., Saute J.A.M., Paranhos Miranda Covaleski A.P., Bulle de Oliveira A.S., Claudino R., Marques W. (2021). Genetic Epidemiology of Familial ALS in Brazil. Neurobiol. Aging.

[9] Wei Q., Zhou Q., Chen Y., Ou R., Cao B., Xu Y., Yang J., Shang H.-F. (2017). Analysis of SOD1 Mutations in a Chinese Population with Amyotrophic Lateral Sclerosis: A Case-Control Study and Literature Review. Sci. Rep..

[10] Oliveira N.A.S., Pinho B.R., Oliveira J.M.A. (2023). Swimming against ALS: How to Model Disease in Zebrafish for Pathophysiological and Behavioral Studies. Neurosci. Biobehav. Rev..

[11] Roggenbuck J., Palettas M., Vicini L., Patel R., Quick A., Kolb S.J. (2020). Incidence of pathogenic, likely pathogenic, and uncertain ALS variants in a clinic cohort. Neurol. Genet..

[12] González-Sánchez M., Ramírez-Expósito M.J., Martínez-Martos J.M. (2025). Pathophysiology, Clinical Heterogeneity, and Therapeutic Advances in Amyotrophic Lateral Sclerosis: A Comprehensive Review of Molecular Mechanisms, Diagnostic Challenges, and Multidisciplinary Management Strategies. Life.

[13] Opie-Martin S., Iacoangeli A., Topp S.D., Abel O., Mayl K., Mehta P.R., Shatunov A., Fogh I., Bowles H., Limbachiya N. (2022). The SOD1-mediated ALS phenotype shows a decoupling between age of symptom onset and disease duration. Nat. Commun..

[14] Rosen D.R., Siddique T., Patterson D., Figlewicz D.A., Sapp P., Hentati A., Donaldson D., Goto J., O’Regan J.P., Deng H.-X. (1993). Mutations in Cu/Zn Superoxide Dismutase Gene Are Associated with Familial Amyotrophic Lateral Sclerosis. Nature.

[15] Deng H.-X., Hentati A., Tainer J.A., Iqbal Z., Cayabyab A., Hung W.-Y., Getzoff E.D., Hu P., Herzfeldt B., Roos R.P. (1993). Amyotrophic Lateral Ssclerosis and Structural Defects in Cu,Zn Superoxide Dismutase. Science.

